# Prevalence of concomitant traumatic cranio-spinal injury: a systematic review and meta-analysis

**DOI:** 10.1007/s10143-018-0988-3

**Published:** 2018-06-07

**Authors:** Mark J. Pandrich, Andreas K. Demetriades

**Affiliations:** University of Edinburgh and Department of Neurosurgery, Western General Hospital, Edinburgh, EH4 2XU UK

**Keywords:** Traumatic brain injury, Traumatic spinal injury, Traumatic cervical spinal injury, Traumatic spinal cord injury, Concomitant cranio-spinal injury

## Abstract

The biomechanical relationship between cranial and spinal structures makes concomitant injury likely. Concomitant cranio-spinal injuries are important to consider following trauma due to the serious consequences of a missed injury. The objective of this review was to estimate the prevalence of concomitant cranio-spinal injury in the adult trauma population. A systematic search of MEDLINE and EMBASE databases to identify observational studies reporting the prevalence of concomitant cranio-spinal injury in the general adult trauma population was conducted on 21 March 2017. The prevalence of concomitant cervical spinal injury in patients with a traumatic brain injury (TBI); the prevalence of concomitant spinal injury in patients with a TBI; the prevalence of concomitant TBI in patients with a cervical spinal injury; and the prevalence of concomitant TBI in patients with a spinal injury were calculated by meta-analysis. Twenty-one studies met the inclusion criteria and were included in this review. The prevalence of concomitant cervical spinal injury in patients with a TBI was found to be 6.5% (95% CI 6.0–7.1%); the prevalence of concomitant spinal injury in patients with a TBI to be 12.4–12.5%; the prevalence of concomitant TBI in patients with a cervical spinal injury to be 40.4% (95% CI 33.0–48.0%); and the prevalence of concomitant TBI in patients with a spinal injury to be 32.5% (95% CI 10.8–59.3%). This review reports the prevalence of concomitant cranio-spinal injury and highlights the importance of considering concomitant injury in patients with a cranial or spinal traumatic injury.

**Electronic supplementary material:**

The online version of this article (10.1007/s10143-018-0988-3) contains supplementary material, which is available to authorized users.

## Introduction

### Trauma and concomitant injury

Head and spinal injury is a disproportionate cause of mortality and morbidity following trauma, with a large burden of disease subsequent to long-term mental and physical disabilities compared to non-neurological traumatic injuries [11, 40, 44].

An impact to the head may result in a traumatic brain injury (TBI), such as haemorrhage, contusions or diffuse injury, resulting in altered brain functioning and a reduced Glasgow coma score (GCS) [25]. Traumatic spinal injury may be defined as fracture or ligamentous damage to the spinal column, damage to the spinal cord or both [10].

Identification and assessment of cranio-spinal injury is a key component of the evaluation of the traumatised patient [1, 40]. Trauma patients frequently have multiple injuries making comprehensive evaluation both difficult and crucial.

### Spinal injury in patients with TBI

The close anatomical and biomechanical association between cranio-spinal structures makes their co-incidence theoretically likely in many trauma patients [14]. Impact to the head is considered to be particularly likely to result in damage to the cervical spine [14]. Spinal injuries are important to consider following trauma as delay in diagnosing an unstable spinal injury may result in neurological deterioration [12, 21]. Altered mental status following a TBI makes the evaluation of possible spinal injury difficult.

### TBI in patients with spinal injury

Spinal injury requires a significant force, often exerted on the head, which may result in a concomitant TBI. Diagnostic findings of TBI may be non-specific, and concomitant TBI is often not detected during the acute management of patients with spinal injury [42]. Concomitant TBI negatively affects the long-term outcome of patients with spinal injury, especially if not identified and treated appropriately [3].

### Rationale for systematic review

The aim of this systematic review and meta-analysis is to estimate the prevalence of concomitant cranio-spinal injuries in the adult trauma population. Studies reporting the prevalence of TBI in patients with spinal injury, and spinal injury in patients with TBI will be reviewed and summarised. It is hoped that estimation of the prevalence of concomitant injury will be of benefit to clinicians in determining the priorities in investigating polytrauma patients.

## Methodology

### Review protocol

Studies investigating the prevalence of TBI in patients with spinal injury and spinal injury in patients with TBI were reviewed. Studies specifically investigating concomitant cervical spinal injury in TBI were also included. Due to the nature of the research question, this was a systematic review of observational studies. Included studies (1) reported the prevalence of concomitant TBI and spinal injury or cervical spinal injury, (2) investigated concomitant injury in a general adult trauma population, and (3) were observational studies. Studies that only reported the prevalence of specific head injuries or only reported the prevalence of specific spinal injuries or only reported concomitant injury secondary to a specific cause, or that reported the prevalence of concomitant injury in a rehabilitation population were excluded. Non-English language publications were also excluded.

### Search strategy

MEDLINE (1946–2017) and EMBASE (1980–2017) databases were searched for this review. Appropriate keywords and MeSH headings relating to spinal injury and traumatic brain injury were identified using PUBMED-PUBREMINER for the database search. These search terms were combined with those relating to study design and population of interest (full search strategy presented in Appendix 1). The database search was conducted on 21 March 2017.

### Study selection

All study selection and screening was carried out by the authors. Duplicate publications were removed and publications screened. Titles and abstracts were reviewed to remove irrelevant studies. A full-text review of remaining publications was conducted. Bibliographies of included publications were reviewed to identify further relevant publications.

### Quality assessment

Quality of reporting was assessed using the STrengthening the Reporting of OBservational studies in Epidemiology (STROBE) checklist for guidance [38]. A risk of bias assessment tool was adapted for use in this study, and studies were deemed to have a low or high risk of bias for each item [22, 29]. Sample size calculations were conducted to determine whether sample size was adequate [13].

### Data extraction

The primary outcome of this review was the prevalence of concomitant injury. Publications were divided into four groups per the prevalence reported: the prevalence of cervical spinal injury in patients with a TBI; the prevalence of spinal injury in patients with a TBI; the prevalence of TBI in patients with a cervical spinal injury; and the prevalence of TBI in patients with a spinal injury. A data extraction sheet was designed and adapted for each group of publications for this purpose.

### Statistical analysis

Statistical analyses were performed using MedCalc, version 15.0. Study heterogeneity was assessed using the *Q* test and *I*^2^ statistic. An *I*^2^ statistic equalling 25, 50, and 75% was interpreted as low, moderate, and high heterogeneity, respectively [20]. Forest plots of prevalence data with 95% confidence intervals were produced for each grouping. The heterogeneity measures were used to inform whether the fixed or random effects model was adopted for meta-analysis. Sub-groups were analysed to identify sources of heterogeneity.

## Results

### Study selection

**Fig. 1:**
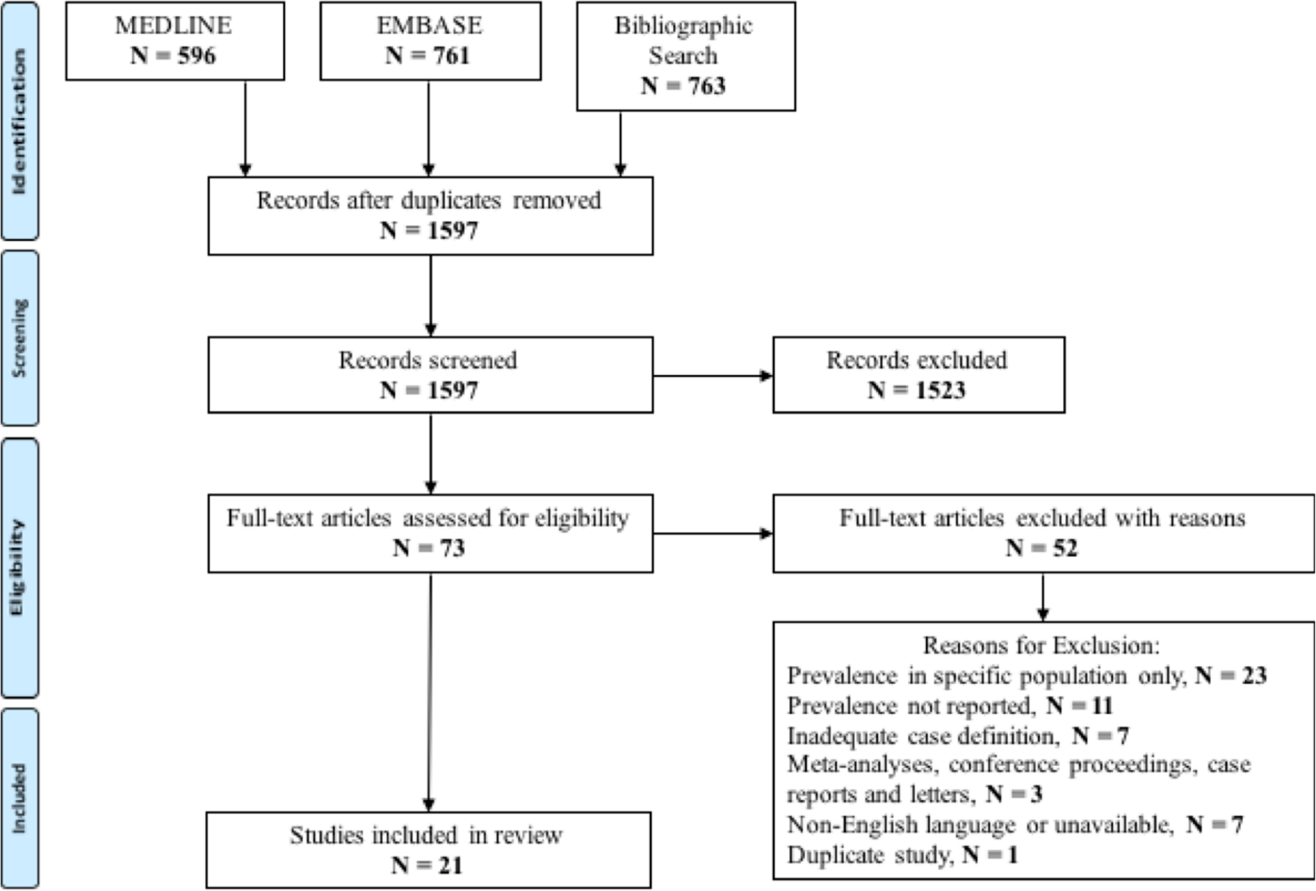
PRISMA flow diagram

The database search identified 1597 publications of which 21 were included in this review, as summarised in the PRISMA flow diagram in Fig. 1. Reasons for exclusion of studies following full-text review were recorded and are presented in Appendix 2.

### Study characteristics

Study characteristics are presented in full in Appendix 3.

All studies included in the review were observational studies either employing cross-sectional or retrospective cohort designs [1, 2, 6, 7, 9, 12, 15, 16, 19, 21, 23, 24, 27, 28, 30, 33–35, 37, 41, 47].

Nine included studies were conducted in the USA [2, 7, 9, 15, 16, 21, 28, 33, 34]; 3 in the UK [1, 12, 19]; 2 in China [41, 47]; and 1 in each of Pakistan [30], Taiwan [6], Australia [27], South Africa [37], Japan [23], Germany [24], and Canada [35]. Studies were not excluded based on publication year. Publication year of included studies ranged from 1988 [33] to 2014 [16].

Studies defined TBI by Glasgow coma score (GCS), Abbreviated Injury Severity (AIS), symptoms at presentation, diagnostic codes, radiological findings, or a combination of these. Spinal injury was defined either by radiological findings or diagnostic codes based on radiological findings.

Studies investigating the prevalence of concomitant injury in a paediatric population were excluded. However, most studies did not specifically exclude paediatric patients [1, 7, 9, 12, 16, 23, 24, 27, 28, 33, 34, 37, 41, 47] and included a limited number of paediatric patients in their analysis. These studies were only included if they took place in adult hospitals/trauma centres or reported the number of paediatric patients included to be minimal.

Most included studies were single-centre studies reporting the prevalence of concomitant injury in admission to a single trauma centre within a set time-period [1, 2, 7, 9, 21, 23, 24, 27, 30, 33, 35, 37, 41, 47]. All other studies included in this review reported the prevalence of concomitant injury from review of a multi-centre trauma database [6, 12, 15, 16, 19, 28, 34].

### Quality assessment of included studies

Results of quality of reporting and risk of bias assessment are presented in Appendix 4.

None of the included studies discussed how the study size was decided or reported using a sample size calculation [1, 2, 6, 7, 9, 12, 15, 16, 19, 21, 23, 24, 27, 28, 30, 33–35, 37, 41, 47]. Only two included studies [7, 19] reported the handling of missing data. None of the included studies discussed how sources of bias were identified and addressed [1, 2, 6, 7, 9, 12, 15, 16, 19, 21, 23, 24, 27, 28, 30, 33–35, 37, 41, 47]. The most significant potential source of bias identified was variable definition of TBI between studies. In some studies, failure to describe the diagnostic criteria and protocol for identification of concomitant injury made assessment of potential bias difficult. Sample size was deemed adequate in all but four studies [1, 24, 33, 47]. None of the included studies calculated a confidence interval for the reported prevalence.

### Cervical spinal injury in TBI

Eleven publications reported the prevalence of concomitant cervical spinal injury in patients with a TBI [2, 12, 15, 21, 27, 28, 30, 33, 34, 37, 41]. The total sample size including all studies reporting this prevalence is 573,870 patients, with sample sizes of individual studies ranging from 265 [37] to 334,864 [28].

**Fig. 2:**
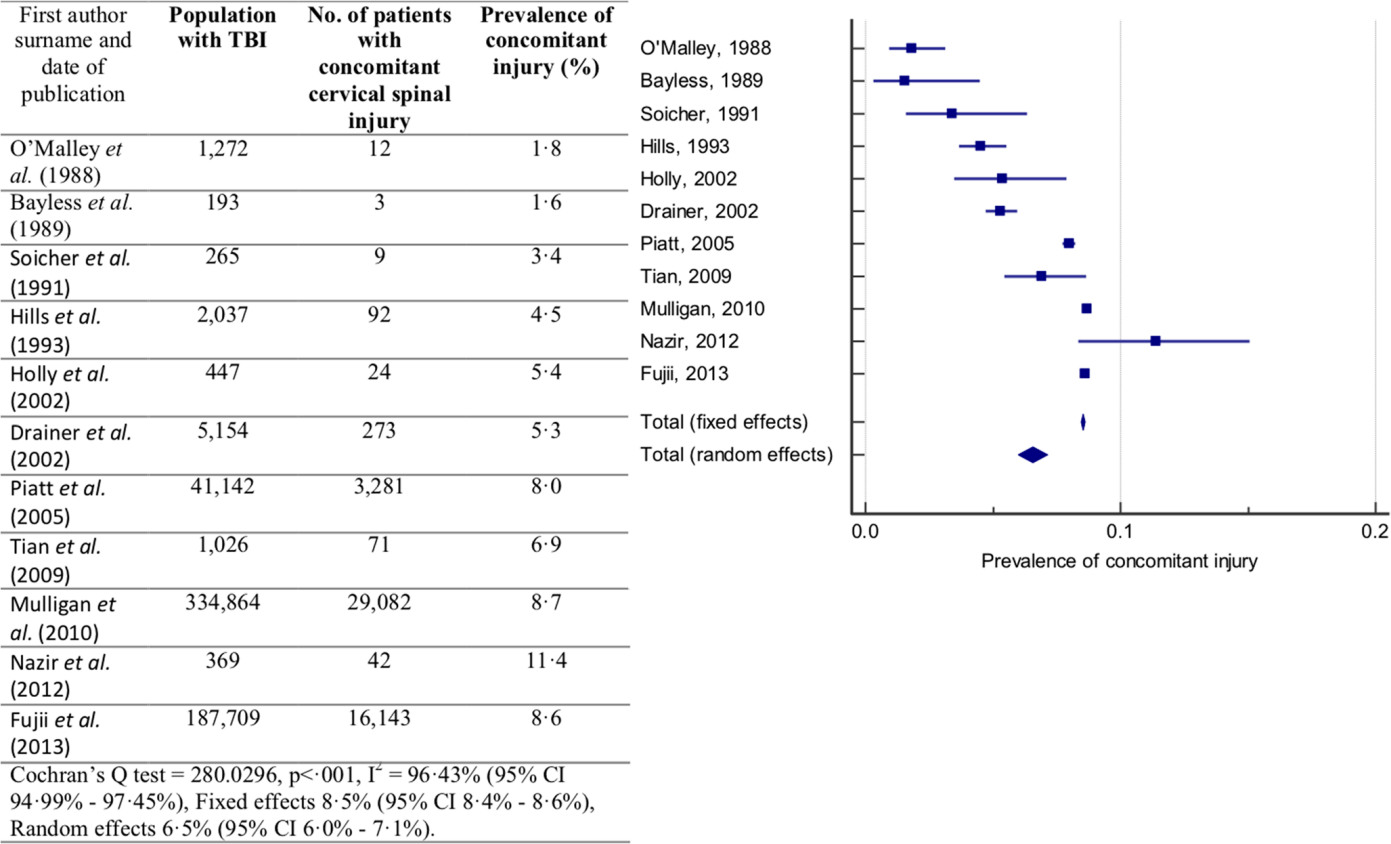
Prevalence of concomitant cervical spinal injury in patients with TBI or head injury: Forest plot of the prevalence of concomitant cervical spinal injury in patients with TBI or head injury with the random effects model used to produce a prevalence estimate

Prevalence of concomitant cervical spinal injury in patients with TBI or head injury reported by each study is presented in Fig. 2. The reported prevalence of concomitant injury ranged from 1.6 [2] to 11.4% [30]. The Cochran’s *Q* statistic indicated that significant heterogeneity between studies (*Q* = 280·0296, *p* < ·001). The *I*^2^ statistic indicated a high-degree of heterogeneity between studies (*I*^2^ = 96.43%; 95% CI 94.99–97.45%). Due to the degree of heterogeneity, the random effects model was used for meta-analysis. The prevalence of concomitant cervical spinal injury was estimated to be 6.5% (95% CI 6.0–7.1%).

**Fig. 3:**
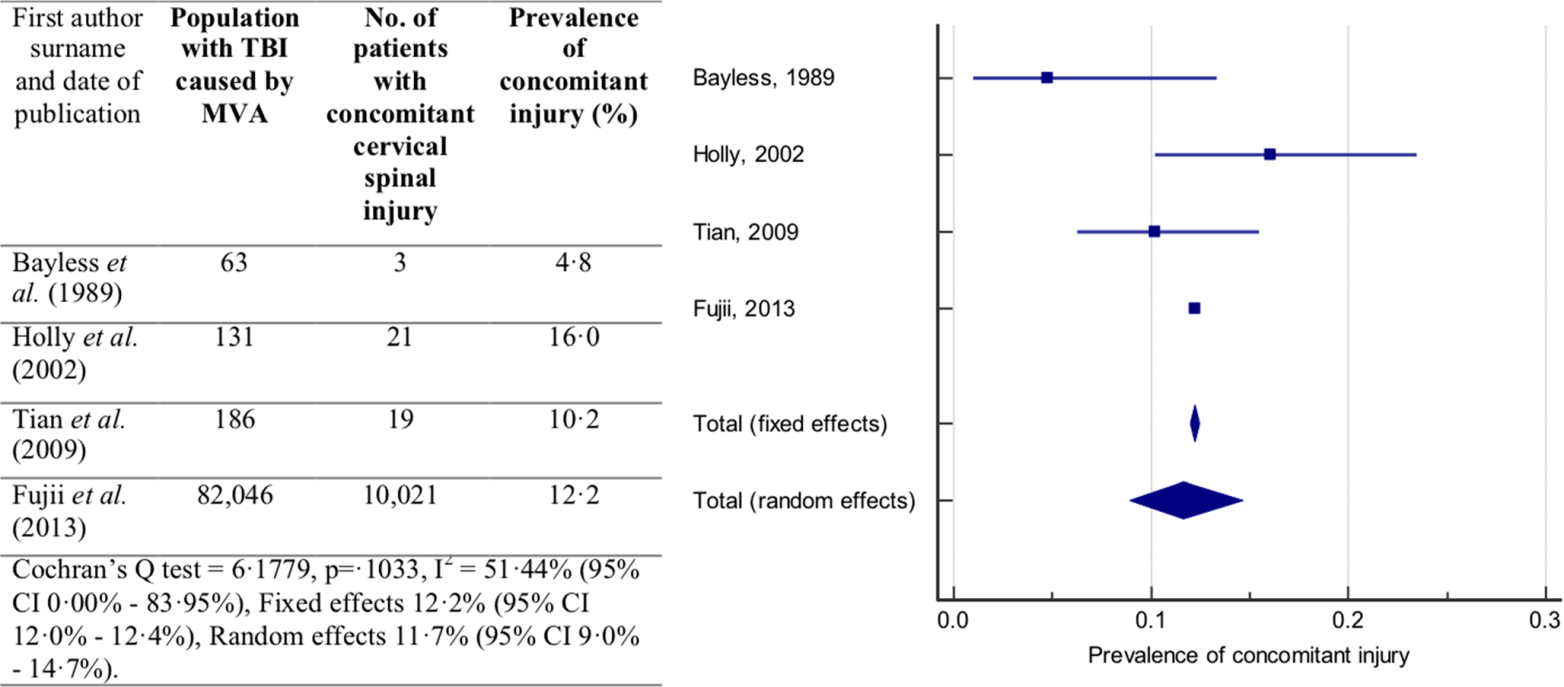
Prevalence of concomitant cervical spinal injury in patients with TBI or head injury. Forest plot of the prevalence of concomitant cervical spinal injury in patients with TBI or head injury caused by a motor vehicle accident (MVA) with the random effects model used to produce a prevalence estimate

Sub-groups were analysed to examine sources of heterogeneity. Examining subgroups of studies that reported the prevalence in patients with GCS 3–12 and GCS less than or equal to 8 did not reduce the heterogeneity. However, four studies reported the prevalence of concomitant cervical spinal injury in patients with TBI or head injury injured in a motor vehicle accident (MVA) [2, 15, 21, 41]. The *I*^2^ statistic indicated that there was only moderate heterogeneity between these studies (*I*^2^ = 51.44%; 95% CI 0.00–83.95%). The random effects model was used for meta-analysis. The prevalence of concomitant cervical spinal injury in patients with a TBI due to a MVA was estimated to be 11.7% (95% CI 9.0–14.7%) and is presented in Fig. 3.

### Spinal injury in TBI

Two studies reported the prevalence of concomitant spinal injury of any spinal level in patients with TBI [15, 16]. Ghobrial et al. [16] had a sample size of 59,832 patients with a reported prevalence of concomitant injury of 12.4%. Fujii et al. [15] had a sample size of 187,709 patients with a reported prevalence of concomitant injury of 12·5%. Meta-analysis was not conducted as only two studies were identified in this category.

### TBI in cervical spinal injury

Nine publications reported the prevalence of concomitant TBI in patients with cervical spinal injury [1, 6, 7, 9, 23, 24, 28, 33, 47]. The total sample size including all studies reporting this prevalence is 69,765 patients, with sample sizes of individual studies ranging from 33 [33] to 58,272 [28] patients.

**Fig. 4:**
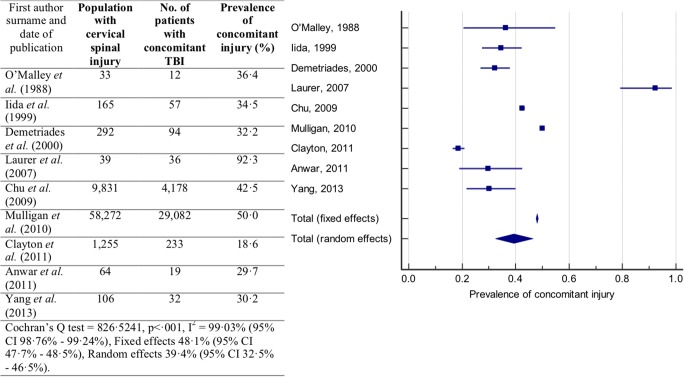
Prevalence of concomitant TBI in patients with a cervical spinal injury. Forest plot of the prevalence of concomitant TBI in patients with a cervical spinal injury with the random effects model used to produce a prevalence estimate

Prevalence of concomitant TBI in patients with cervical spinal injury reported by each study is presented in Fig. 4. The reported prevalence of concomitant injury ranged from 18.6 [7] to 92.3% [24]. The Cochran’s *Q* statistic indicated that there was significant heterogeneity between studies (*Q* = 795.4110, *p* < .001). The *I*^2^ statistic indicated a high-degree of heterogeneity between studies (*I*^2^ = 99.12%; 95% CI 98.86–99.32%). The random effects model was used for meta-analysis. The prevalence of concomitant TBI was found to be 40.4% (95% CI 33.0–48.0%).

Studies did not report sufficient detail of severity of TBI or mechanism of injury to allow for sub-group analysis to investigate heterogeneity.

### TBI in spinal injury

Five publications reported the prevalence of concomitant TBI in patients with all spinal injury, reporting the prevalence of TBI in patients with an injury at any spinal level [1, 6, 19, 24, 35]. The total sample size including all studies reporting this prevalence is 92,780 patients, with sample sizes of individual studies ranging from 183 [24] to 51,541 [6] patients.

**Fig. 5:**
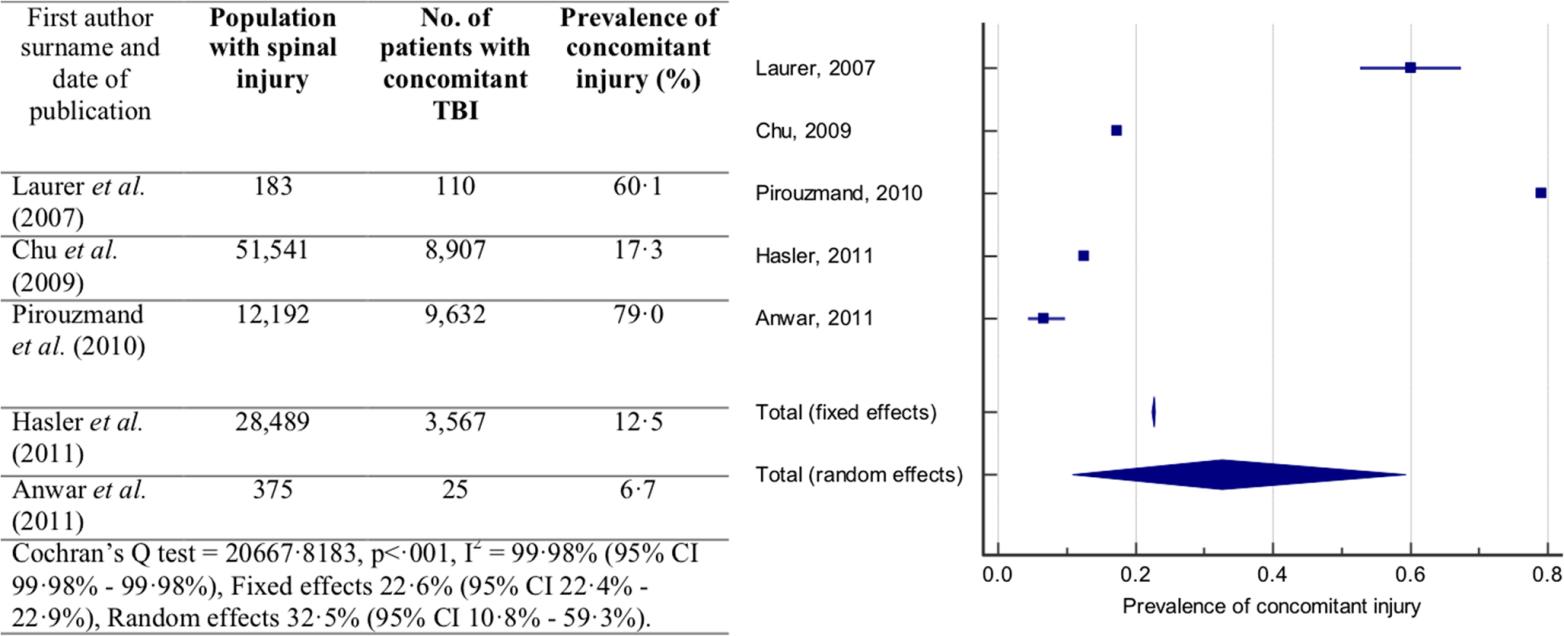
Prevalence of concomitant TBI in patients with a spinal injury. Forest plot of the prevalence of concomitant TBI in patients with a spinal injury with the random effects model used to produce a prevalence estimate

Prevalence of concomitant TBI in patients with spinal injury reported by each study is presented in Fig. 5. The reported prevalence of concomitant injury ranged from 6.7 [1] to 79.0% [35]. The Cochran’s *Q* statistic indicated that there was significant heterogeneity between studies (*Q* = 20,667.8183, *p* < .001). The *I*^2^ statistic indicated a high-degree of heterogeneity between studies (*I*^2^ = 99.98%; 95% CI 99.98–99.98%). The random effects model was used for meta-analysis. The prevalence of concomitant TBI was found to be 32.5% (95% CI 10.8–59.3%).

Studies did not report sufficient detail of severity of TBI or mechanism of injury to allow for sub-group analysis to investigate heterogeneity.

## Discussion

### Summary of findings

The prevalence of concomitant injury in several patient groups is reported. The prevalence of concomitant cervical spinal injury in patients with TBI was estimated to be 6.5%. The prevalence of concomitant cervical spinal injury in patients with TBI injured in a MVA was estimated to be 11.7%. Two studies reported the prevalence of concomitant spinal injury of any location in patients with TBI to be 12.5 [15] and 12.4% [16].

The prevalence of concomitant TBI in patients with cervical spinal injury was estimated to be 40.4%. The prevalence of concomitant TBI in patients with spinal injury of any location was estimated to be 32.5%.

### Impact of methodology on prevalence estimates

Significant true heterogeneity is expected in a review of epidemiological studies due to diversity of study designs and populations [39]. To allow for meaningful quantitative synthesis of prevalence estimates, attempts were made to reduce this heterogeneity. Inclusion and exclusion criteria were designed to ensure included studies were comparable. All included studies reported the prevalence of concomitant cranio-spinal injury in the general adult trauma population irrespective of patient outcome. For example, studies investigating the prevalence of concomitant cranio-spinal injury in a rehabilitation population were excluded [3, 4, 26, 36, 46], as patients who died or were lost to follow-up would not be included in the prevalence estimated. Similarly, studies investigating prevalence of concomitant cranio-spinal injury in an autopsy population were excluded as they would not include patients who had survived [17, 31, 43].

Despite these efforts, the *I*^2^ statistic indicated a high degree of true heterogeneity in all groups. In combining the prevalence estimates of studies, several assumptions were made that may account for this heterogeneity. Sub-group analysis was conducted to investigate heterogeneity [18].

Several studies suggest that mechanism of injury determines the risk of concomitant injury [2, 15, 21, 30]. Therefore, variation in incidence of different mechanisms of injury between studies may account for the observed heterogeneity in this review. For example, as studies were not excluded based on country of origin or year of publication, it is likely that the incidence and severity of MVAs varies between studies [45]. Interestingly, analysing the prevalence of concomitant cervical spinal injury in patients with TBI caused by MVA, as a sub-group, reduced the heterogeneity between studies and increased the estimated prevalence. This supports the hypothesis that MVAs are particularly likely to cause concomitant injury and suggests that differing incidence in mechanisms of injury is a source of heterogeneity in this review. Due to inconsistent categorisation and insufficient reporting of severity of injury, type of injury, and mechanism of injury, this review was unable to further investigate risk factors for concomitant cranio-spinal injury.

The variable definition of TBI could impact estimates of prevalence of concomitant injury. As many studies included in this review used reduced GCS as an indicator of significant head injury, the influence of TBI definition was investigated by analysing the prevalence of concomitant cervical spinal injury in patients with GCS 3–12 and GCS ≤ 8. However, heterogeneity was not reduced in these sub-groups indicating that definition of TBI alone may not be the cause of heterogeneity in this review.

Many studies inadequately reported the protocol used to assess for spinal injury. Protocols were especially poorly reported by studies of national databases. Among studies that did report a protocol all initially assessed the cervical spine with plain radiography. Except for some older studies [2, 33, 37], all reported protocols also involved either routine, or clinical suspicion dependent CT-scanning of the cervical spine. Only two studies reported protocols with the option of MRI scan [12, 30]. The necessary radiographic evaluation of the cervical spinal, particularly, is an unresolved and controversial issue. Plain radiography is often considered to be insufficiently sensitive to exclude spinal injury, especially as it is often of unsatisfactory quality in acute trauma [34]. CT-scanning of the cervical spinal can be performed during the initial head scan, but concerns remain about the detection of purely ligamentous injuries and isolated spinal cord injury [5]. MRI can demonstrate ligamentous and intrinsic spinal cord injury, but is a lengthy procedure and magnetic fields are an obstacle to physiological monitoring [8]. It is possible that studies that did not use protocols involving plain radiography, CT-scanning, and MRI underestimated the prevalence of concomitant injury.

### Significance of findings

In the initial management of a cranially or spinally injured patient, the possibility of further cranio-spinal injuries must be considered. The prevalence estimates presented in this review may be used to inform a judgement of the likelihood of such concomitant injuries to allow investigative and management priorities to be decided; allowing the risks of a missed spinal injury to be balanced with the risks of frustrating the investigation of other critical injuries [33].

The finding that 6.5% of patients with TBI have a cervical spinal injury supports the high priority that the possibility of cervical spinal injury is given in Advanced Trauma Life Support protocols [32]. The prevalence of spinal injury in trauma patients without TBI was not investigated in this review; however, this has previously reported to be significantly lower than 6.5% [27]. This review also found that around half of spinal injuries in patients with TBI occur in the cervical region, which is consistent with the view that the cervical spine is particularly vulnerable to damage following impact to the head [14].

The results of this review suggest that concomitant TBI in spinal injury is more common than concomitant spinal injury in TBI. The high prevalence of concomitant TBI in patients with cervical spinal injury or spinal injury of any location, 40.4 and 32.5% respectively, suggests that a concomitant TBI is very likely in any patient with a traumatic spinal injury.

## Conclusion

The prevalence of concomitant cranio-spinal injury reported in this study emphasises the need to consider spinal injury in all patients with TBI and TBI in all patients with spinal injury. Further research is required to identify risk factors for concomitant injury and to update prevalence estimates to reflect revised protocols for the clearance of the spine following traumatic injury.

## Electronic supplementary material

ESM 1 (DOCX 66 kb)

ESM 2 (DOCX 115 kb)

ESM 3 (DOCX 104 kb)

ESM 4 (DOCX 101 kb)
