# Supplementary material for: Prevalence of concomitant traumatic cranio-spinal injury: a systematic review and meta-analysis
Source: Neurosurg Rev. 2018 Jun 7;43(1):69–77. doi: 10.1007/s10143-018-0988-3 (PMC7010651; doi:10.1007/s10143-018-0988-3)
Supplement: Supplementary file 1 — (DOCX 66 kb) [file 10143_2018_988_MOESM1_ESM.docx]

Appendix 1: Full Search Strategy

Database searches conducted: 21/3/2017

Medline search terms: ((spinal cord injuries/ or central cord syndrome) OR (spinal injuries/ or spinal fractures/) OR (cervical vertebrae/ in [injuries] OR ((spin* or cervical) adj2 (fracture* or injur* or wound* or trauma*)).ti,ab)) AND ((exp Craniocerebral Trauma/) OR ((head or brain or skull)adj(injur* or trauma* or fracture*)).ti.ab)) AND ((incidence/ or prevalence) OR (risk/or risk factors) OR (epidemiology/) OR (risk* or incidence or prevalence).ti,ab.), limit to all adult (19 plus years)

Embase search terms: ((spinal cord injuries/ or central cord syndrome/) OR (spinal injuries/ or spinal fractures/) OR (cervical spine injury/) OR ((spine* or cervical) adj2 (fracture* or injur* or wound* or trauma*)).ti,ab.)) AND ((exp Craniocerebral Trauma/) OR ((head or brain or skull)adj(injur* or trauma* or fracture*)).ti.ab)) AND ((incidence/ or prevalence) OR (risk/or risk factors) OR (epidemiology/) OR (risk* or incidence or prevalence).ti,ab.), limit to (adult <18yo to 64 years> or aged <65+ years>)

“*cervical vertebrae/” was replaced with “cervical spine injury” for the EMBASE search.*
