# Supplementary material for: Prevalence of concomitant traumatic cranio-spinal injury: a systematic review and meta-analysis
Source: Neurosurg Rev. 2018 Jun 7;43(1):69–77. doi: 10.1007/s10143-018-0988-3 (PMC7010651; doi:10.1007/s10143-018-0988-3)
Supplement: Supplementary file 2 — (DOCX 115 kb) [file 10143_2018_988_MOESM2_ESM.docx]

| *Appendix 2: Excluded studies with reasons* | |
| --- | --- |
| First author surname and date of publication | **Reason for exclusion** |
| Aaland *et al.* (1996) | Only reports prevalence of injuries in patients with missed diagnosis. |
| Ackland *et al.* (2006) | Review article |
| Anekstein *et al.* (2008) | Does not report prevalence of concomitant injury. |
| Bardon *et al.* (2012) | Only patients that have undergone whole-body CT and does not report on head injury only patients with significantly abnormal CTs. |
| Barret *et al.* (2009) | Focussed on non-trauma related incidental findings so does not report prevalence of concomitant injury. |
| Berne *et al.* (1999) | Mixes head injury with alcohol intoxication as reason to be unreliable. |
| Blackmore *et al.* (1999) | Not available |
| Bloom *et al.* (1997) | Only reports on patients with occipital condyle fractures. |
| Bombardier *et al.* (2016) | Assessment of rehabilitation patients only. |
| Brohi *et al.* (2005) | Only intubated patients included. |
| Budisin *et al.* (2016) | Reviews patients 3-6 months post-injury in rehab rather than on admission. |
| Chang *et al.* (2005) | Only evaluates distracting injuries in patients with normal mental status. |
| Chiu *et al.* (2001) | Does not specify reason for reduced GCS, some intoxicated etc. |
| Davidoff *et al.* (1985) | Not available. |
| Davidoff *et al.* (1988) | Not available. |
| Demetriades *et al.* (2012) | Not primary research. Comment on journal article. |
| Diaz *et al.* (2003) | Does not specify reason for reduced GCS, some intoxicated etc. |
| Dunae *et al.* ((2007) | Analysis of subgroup of blunt trauma patients with GCS 15. |
| Dunae *et al.* (2011) | Analysis of accuracy of investigation. Does not report prevalence. |
| Dunae *et al.* (2011) | Analysis of accuracy of investigation. Does not report prevalence. |
| Dunae *et al.* (2016) | Analysis of accuracy of investigation. Does not report prevalence. |
| Gbaanador *et al.* (1986) | Does not report prevalence of cervical spine injury in general adult trauma population. |
| Gonazalez *et al.* (2009) | Does not specify reason for reduced GCS, some intoxicated etc. |
| Griffin *et al.* (1985) | Does not present prevalence of head injury in spinal injury group. |
| Griffin *et al.* (1985) | Only presents prevalence of head injury in mortality group. |
| Hagen *et al.* (2010) | Only spinal cord injury assessed rather than all spinal injuries. |
| Hasler *et al.* (2012) | Significant number of patients with missing GCS data. |
| Hogan *et al.* (2005) | Does not give detail of head injuries. |
| Ivanov *et al.* (2007) | not available in English |
| Kach *et al.* (1993) | not available in English |
| Karacan *et al.* (2000) | Only presents data for spinal cord injury. |
| Karamehmetoglu *et al.* (1995) | Insufficient detail on prevalence of head injury. |
| Karamehmetoglu *et al.* (1997) | Insufficient detail on prevalence of head injury. |
| Lefering *et al.* (2008) | Cervical spinal injury included in head injury group. |
| Leong *et al.* (2013) | Prevalence of spinal injury not specifically reported. |
| Macciocchi *et al.* (2008) | Only examines patients in rehab. |
| Malomo *et al.* (1995) | Not available. |
| Martins *et al.* (1998) | Only assesses spinal cord injury |
| Michael *et al.* (1989) | Insufficient data presented for inclusion in meta-analysis. |
| Mulligan *et al.* (2010) | Only reports prevalence in patients with craniomaxillofacial fractures. |
| Neumann *et al.* (2009) | Prevalence of head injury not presented in whole group (only in mortality group). |
| Padayachee *et al.* (2006) | Comparison of investigations does not report prevalence. |
| Piatt *et al.* (2005) | Repetition of study already included, published in different journal. |
| Prasad *et al.* (1999) | Does not report exact prevalence of head injury in spinal injury group. |
| Rao *et al.* (1969) | Not available. |
| Rosi *et al.* (2012) | Only investigates car accidents. |
| Rosso *et al.* (2007) | Only prevalence of cord injury. |
| Schenarts *et al.* (2001) | Only reports on upper cervical spinal injuries |
| Selvarajah *et al.* (2013) | Spinal cord injury only. |
| Sharma *et al.* (2014) | Rehabilitation population. |
| Stanislas *et al.* (1998) | Only reports thoracolumbar spinal injuries. |
| Thesleff *et al.* (2017) | Only reports incidence of spinal fracture rather than all spinal injuries. |
| Varma *et al.* 2010) | Analysis only of mortality group. |
| Vahldiek *et al.* (2016) | Only includes patients with ‘blunt minor trauma’: not general adult trauma population. |
| Widder *et al.* (2004) | Comparison of sensitivities of investigations. |
| Williams *et al.* (1992) | Age of included patients not specified. |
| Wilmot *et al.* (1985) | Study only of patients admitted to rehabilitation unit. |
| Yang *et al.* (2016) | Only spinal cord injury assessed rather than all spinal injuries. |
