# Supplementary material for: Prevalence of concomitant traumatic cranio-spinal injury: a systematic review and meta-analysis
Source: Neurosurg Rev. 2018 Jun 7;43(1):69–77. doi: 10.1007/s10143-018-0988-3 (PMC7010651; doi:10.1007/s10143-018-0988-3)
Supplement: Supplementary file 3 — (DOCX 104 kb) [file 10143_2018_988_MOESM3_ESM.docx]

Appendix 3: Study Characteristics

Study Characteristics. *Table 1:* Characteristics of studies reporting prevalence of cervical spine injury in TBI. *Table 2:* Characteristics of studies reporting prevalence of spinal injury in TBI. *Table 3:* Characteristics of studies reporting prevalence of TBI in cervical spine injury. *Table 4:* Characteristics of studies reporting prevalence of TBI in spinal injury

| *Table 1: Characteristics of studies reporting prevalence of cervical spinal injury in TBI (sorted by publication date)* | | | | | |
| --- | --- | --- | --- | --- | --- |
| First author surname and date of publication | **Place, Time of Study, Study design and Sampling frame** | **Aim** | **Population** | **Case definition (TBI)** | **Identification of cervical spinal injury** |
| O’Malley *et al.* (1988) | 1. USA 2. 1986 – 1987 3. Cross-sectional 4. Consecutive admission to level 1 regional trauma center. | Identify the risk of c-spine injury in head injured patients. | 664 patients with head trauma. | Patients admitted with head injury and AIS of ≥2. | 3-view cervical spine series with CT if clinically indicated. |
| Bayless *et al.* (1989) | 1. USA 2. 1983 – 1983 3. Retrospective Cohort 4. Consecutive admission to county hospital. | Establish an incidence of cervical spine injuries in significant blunt head trauma. | 193 patients with blunt head trauma >12 years old. | Blunt head trauma defined as observed LOC, undocumented LOC with retrograde amnesia or any other blunt head trauma with or without LOC deemed significant by consulting service. | Plain radiographs of cervical spine (cross-table, lateral, AP, odontoid).  Flexion-extension and oblique not routinely performed. |
| Soicher *et al.* (1991) | 1. South Africa 2. 1990 – 1990 3. Retrospective cohort study 4. Admissions to hospital. | Determine the prevalence of c-spine injury in patients with head injury. | 265 patients with severe head injury. | RTAs or falls from a height with head injury with LOC, GCS <12 or skull fracture. | Plain cervical radiographs (AP and lateral views) + CT in appropriate cases. |
| Hills *et al.* (1993) | 1. Australia. 2. 1987 – 1991 3. Cross-sectional 4. Admissions to hospital. | Establish possible association of cervical spine injuries with craniocerebral and facial injuries. | 2,037 patients with head injuries. | Head injuries with AIS scores of ≥2. | Initial emergency room roentgenograms, CT or Post-mortem examination. |
| Holly *et al.* (2002) | 1. USA 2. 1992 – 1996 3. Cross-sectional 4. Admissions with TBI to brain injury centre. | Define incidence of cervical injury associated with moderate or severe brain injury and identify high-risk patients. | 447 patients with moderate or severe head injury >18 years old. | Patients with head injury and GCS >12 (moderate) or GCS 3-12 (severe). | Plain radiographs of cervical spine (lateral, AP and odontoid) + CT of occiput-C2 and cervicothoracic junction if poorly visualised + CT of suspicious areas.  Lateral flexion-extension radiography to rule out ligamentous instability 2-3 days post-injury. |
| Drainer *et al.* (2002) | 1. UK 2. 1995 – 2000 3. Cross-sectional 4. Scottish Trauma Audit Group (STAG) database records. | Evaluate the incidence of CSI in patients sustaining blunt head injuries in a Scottish population. | 5,154 head-injured patients. | All patients coded as having head-injury. | Protocol not reported. Identified with plain radiography, CT, MRI and autopsy examination. |
| Piatt *et al.* (2005) | 1. USA 2. 1986 – 2004 3. Cross-sectional 4. Pennsylvania Trauma Outcomes Study (PTOS) database records. | Determine traumatic brain injury patients at very low risk of concomitant cervical spine injury. | 41,142 patients with TBI. | Patients with TBI and GCS ≤8 on admission. | Patients coded as having cervical spine injury, including fracture with or without spinal cord injury, dislocation and isolated spinal cord injury.  Protocol for identification not reported. |
| Tian *et al.* (2009) | 1. China 2. 1999 – 2003 3. Cross-sectional 4. Admissions to neurosurgery department. | Determine the incidence and risk factors for concomitant cervical injury in comatose patients with traumatic brain injury (TBI). | 1,026 patients with TBI. | Patients with TBI and slight coma (GCS 9-12), middle coma (GCS 6-8) and deep come (GCS 3-5). | Plain radiographs of cervical spine (AP, lateral, bilateral oblique, odontoid) + CT if clinical suspicious or poor visualisation. |
| Mulligan *et al.* (2010) | 1. USA 2. 2002 – 2006 3. Cross-sectional 4. National Trauma Data Bank records. | Review the incidence of facial fractures, c-spine injuries, and head injuries at trauma centers across the United States. | 334,864 records of patients with head injury. | All patients with ICD-9 diagnostic codes relating to head injury (skull fracture, brain contusions or lacerations, intracranial haemorrhage, concussion with LOC). | ICD-9 diagnostic codes relating to spinal injury (fracture, dislocation, fracture-dislocation). |
| Nazir *et al.* (2012) | 1. Pakistan. 2. 2011 – 2012 3. Cross-sectional 4. Admissions to neurosurgery department. | Determine the frequency of cervical injury in patients with moderate to severe head injury and different types of trauma. | 269 patients with moderate to severe head injury >2 years old. | Patients with head injury and GCS 9-12 (moderate) or GCS ≤8. | Plain radiographs, CT scan and MRI of cervical spine. |
| Fujii *et al.* (2013) | 1. USA 2. 2007 - 2007 3. Cross-sectional 4. National Trauma Data Bank records | To describe potential risk factors associated with CSI among trauma patients with TBI. | 187,709 patients with TBI >18 years old. | TBI defined as bump, blow or jolt to the head or a penetrating head injury that disrupts the normal functioning of the brain.  Mild (GCS 13-15), moderate (GCS 9-12) and severe (GCS ≤8) | Patients coded as having cervical spine injury, including spine fracture and/or dislocation.  Protocol for identification not reported. |

| *Table 2: Characteristics of studies reporting prevalence of spinal injury in TBI (sorted by publication date)* | | | | | |
| --- | --- | --- | --- | --- | --- |
| First author surname and date of publication | **Place, Time of Study, Study design and Sampling frame** | **Aim** | **Population** | **Case definition (TBI)** | **Identification of spinal injury** |
| Fujii *et al.* (2013) | 1. USA 2. 2007 – 2007 3. Cross-sectional 4. National Trauma Data Bank records. | Examination of risk factors for CSI among patients with TBI. | 187,709 patients with TBI. | TBI defined as bump, blow or jolt to the head or a penetrating head injury that disrupts the normal functioning of the brain.  Mild (GCS 13-15), moderate (GCS 9-12) and severe (GCS ≤8) | Patients coded as having spine fracture or dislocation. |
| Ghobrial *et al.* (2014) | 1. USA 2. 2008 - 2008 3. Cross-sectional 4. The Healthcare Cost and Utilization Project (HCUP), National Inpatient Sample (NIS) database records | Define the incidence of concurrent RTBI and SCI. | 59,832 patients with TBI. | Patients coded as having TBI, including brain injury and skull fractures. | Patients coded as having spinal injury including spinal fractures with and without spinal cord injury and spinal cord injury without bony injury.  Protocol for identification not reported. |

| *Table 3: Characteristics of studies reporting prevalence of TBI in cervical spine injury (sorted by publication date)* | | | | | |
| --- | --- | --- | --- | --- | --- |
| First author surname and date of publication | **Place, Time of Study, Study design and Sampling frame** | **Aim** | **Population** | **Case definition (Cervical spine injury)** | **Definition of TBI used** |
| O’Malley *et al.* (1988) | 1. USA 2. 1986 – 1987 3. Cross-sectional 4. Consecutive admissions to level 1 trauma center. | Identify the risk of c-spine injury in head injured patients. | 33 patients with c-spine secondary to blunt trauma. | Cervical spine fracture or dislocation identified by radiography with AIS ≥2. | Significant head injury defined with AIS ≥2 and loss of consciousness. |
| Iida *et al.* (1999) | 1. Japan 2. Not reported (published 1999) 3. Cross-sectional 4. Neurosurgery department patients undergoing radiography. | Determine the link between cervical spine and/or spinal cord injuries and head trauma. | 188 patients with cervical spine injury, spinal cord injury or both. | Cervical spine fracture or dislocation identified by radiography. | Patients with head injury and GCS 13-15 (mild), GCS 9-12 (moderate) and GCS 3-8 (severe). |
| Demetriades *et al.* (2000) | 1. USA 2. 1993 – 1997 3. Cross-sectional 4. Admissions to county hospital. | Investigate the incidence and type of cervical spine trauma according to mechanism of injury. | 292 patients with cervical spine injury. | Patients with cervical spine fracture, subluxations and/or spinal cord injuries. | Patients with head injury and GCS 3-12. |
| Laurer *et al.* (2007) | 1. Germany 2. 2003 – 2006 3. Cross-sectional 4. Admissions to emergency department. | Evaluate the extent and distribution of spinal injury in patients suffering from severe multiple injuries. | 39 patients with cervical injury and ISS >15. | Radiographically identified cervical spine injury. | Head injury with AIS ≥1 |
| Chu *et al.* (2009) | 1. Taiwan 2. 2000 – 2003 3. Cross-sectional 4. Taiwan’s National Health Insurance database records. | Investigate the prevalence of concomitant injuries among hospitalised acute spinal trauma patients aged 20 and over and the effects of those injuries on medical utilisation in Taiwan. | 9,831 patients with cervical spinal injuries >20 years old. | Fractures of the vertebral column with or without spinal cord injury and isolated spinal cord injury. | Head trauma defined as fracture of the skull and intracranial injury. |
| Mulligan *et al.* (2010) | 1. USA 2. 2002 – 2006 3. Cross-sectional 4. National Trauma data bank records. | Review the incidence of facial fractures, c-spine injuries, and head injuries at trauma centers across the United States. | 58,262 patients with cervical spine injuries. | ICD-9 diagnostic codes, including fracture, dislocation and spinal cord injury in the cervical region. | ICD-9 diagnostic codes. |
| Clayton *et al.* (2011) | 1. USA 2. 1997 – 2002 3. Cross-sectional 4. Admission to level 1 trauma centre. | Identify predictor of cervical spine injury. | 1,255 patients with cervical spine injury. | Patients diagnosed with injury to cervical spine, including dislocation, fractures, fractures with cord injury and isolated cord injury as recorded in medical notes. | Head injury ICD-9 diagnostic codes. |
| Anwar *et al.* (2011) | 1. UK 2. 2006 – 2009 3. Cross-sectional 4. Admission to Spinal Cord Injury Centre | Determine incidence of associated injuries with spinal cord trauma and spinal injury. | 64 patients with cervical spine injuries. | Radiographically determined cervical spine injury. | Diagnosed head injury, not further defined. |
| Yang *et al.* (2013) | 1. China 2. 2011 – 2013 3. Cross-sectional 4. Admissions to Orthopaedic hospital. | Describe the epidemiology of c-spine injury. | 106 patients with cervical spine injuries. | Cervical fracture with or without cervical cord injury diagnosed by plain radiographs in two plains with CT scans where indicated. | Head injury, not further defined. |

| *Table 4: Characteristics of studies reporting prevalence of TBI in spinal injury (sorted by publication date)* | | | | | |
| --- | --- | --- | --- | --- | --- |
| First author surname and date of publication | **Place, Time of Study, Study design and Sampling frame** | **Aim** | **Population** | **Case definition (spinal injury)** | **Definition of TBI** |
| Laurer *et al.* (2007) | 1. Germany 2. 2003 – 2006 3. Cross-sectional 4. Admissions to emergency department. | Evaluate the extent and distribution of spinal injury in patients suffering from severe multiple injuries. | 183 patients with spinal injury and ISS >15. | Radiographically identified spinal injury. | Head injury with AIS ≥1 |
| Chu *et al.* (2009) | 1. Taiwan 2. 2000 – 2003 3. Cross-sectional 4. Taiwan’s National Health Insurance database records. | Investigate the prevalence of concomitant injuries among hospitalised acute spinal trauma patients aged 20 and over and the effects of those injuries on medical utilisation in Taiwan. | 51,541 patients with spinal injuries >20 years old. | Fractures of the vertebral column with or without spinal cord injury and isolated spinal cord injury. | Head trauma defined as fracture of the skull and intracranial injury. |
| Pirouzmand *et al.* (2010) | 1. Canada 2. 1986 – 2006 3. Cross-sectional 4. Admissions to level 1 trauma center. | Examine epidemiology of spine and spinal cord injuries. | 12,192 patients with spinal injury and an ISS >12. | Any spinal fracture and/or dislocation with or without SCI. | Head injury, not further defined. |
| Hasler *et al.* (2011) | 1. UK 2. 1988 – 2009 3. Cross-sectional 4. Trauma Audit and Research Network database records. | Determine predictors of spinal trauma. | 28,489 patients with spinal trauma. | Patients with spinal injury requiring hospital admission >72 hours, or require HDU or ICU care, or die because of their injuries. | Head injury with AIS ≥3. |
| Anwar *et al.* (2011) | 1. UK 2. 2006 – 2009 3. Cross-sectional 4. Admission to Spinal Cord Injury Centre | Determine incidence of associated injuries with spinal cord trauma and spinal injury. | 375 patients with spinal injuries. | Radiographically determined spine injury. | Diagnosed head injury, not further defined. |
