# Supplementary material for: Prevalence of concomitant traumatic cranio-spinal injury: a systematic review and meta-analysis
Source: Neurosurg Rev. 2018 Jun 7;43(1):69–77. doi: 10.1007/s10143-018-0988-3 (PMC7010651; doi:10.1007/s10143-018-0988-3)
Supplement: Supplementary file 4 — (DOCX 101 kb) [file 10143_2018_988_MOESM4_ESM.docx]

| *Appendix 4: Quality of Reporting and Risk of Bias Assessment* | |
| --- | --- |
| STROBE checklist | |
| Item | **Number of studies fulfilling criteria / total number of studies** |
| 1. a) Indicate the study's design with a commonly used term in the title or the abstract. | 4 / 21 |
| 1. b) Provide in the abstract an informative and balanced summary of what was done and what was done and what was found. | 19 / 21 |
| 2. Explain the scientific background and rationale for the investigation being reported. | 21 / 21 |
| 3. State specific objectives, including any prespecified hypotheses. | 21 / 21 |
| 4. Present key elements of study design early in the paper. | 20 / 21 |
| 5. Describe the setting, locations and relevant dates, including periods of recruitment, exposure, follow-up, and data collection. | 20 / 21 |
| 6. a) Cohort study- give the eligibility criteria, and the sources and methods of selection of participants. Describe methods of follow-up. Case-control study- Give the eligibility criteria, and the sources and methods of case ascertainment and control selection. Give the rationale for the choice of cases and controls. Cross-sectional study- give the eligibility criteria, and the sources and methods of selection of participants. | 19 / 21 |
| 6. b) Cohort study- for matched studies, give matching criteria and number of exposed and unexposed. Case-control study- for matched studies, give matching criteria and the number of controls per case. | n/a |
| 7. Clearly define all outcomes, exposures, predictors, potential confounders, and effect modifiers. Give diagnostic criteria, if applicable. | 18 / 21 |
| 8. For each variable of interest, give sources of data and details of methods of assessment (measurement). Describe comparability of assessment methods if these is more than one group. | 12 / 21 |
| 9. Describe any efforts to address potential sources of bias. | 0 / 21 |
| 10. Explain how the study size was arrived at. | 0 / 21 |
| 11. Explain how quantitative variables were handled in the analyses. If applicable, describe which groupings were chosen and why. | 20 / 21 |
| 12. a) Describe all statistical methods, including those used to control for confounding. | 13 / 21 |
| 12. b) Describe any methods used to examine subgroups and interactions. | 19 / 21 |
| 12. c) Explain how missing data were addressed. | 2 / 21 |
| 12. d) Cohort study- If applicable, explain how loss to follow-up was addressed. Case-control study- if applicable, explain how matching of cases and controls was addressed. Cross-sectional study- if applicable, describe analytical methods taking account of sampling strategy. | n/a |
| 12. e) Describe any sensitivity analyses. | 0 / 21 |
| 13. a) Report numbers of individuals at each stage of study- e.g. numbers potentially eligible, examined for eligibility, confirmed eligible, included in the study, completing follow-up, and analysed. | 16 / 21 |
| 13. b) Give reasons for non-participation at each stage. | 10 / 21 |
| 13. c) Consider use of a flow diagram. | 3 / 21 |
| 14. a) Give characteristics of study participants (e.g. demographic, clinical, social) and information on exposures and potential confounders. | 17 / 21 |
| 14. b) Indicate number of participants with missing data for each variable of interest. | 3 / 21 |
| 14. c) (cohort only) Summarise follow-up time (e.g. average and total amount) | n/a |
| 15. Cohort study- Report numbers of outcome events or summary measures over time. Case-control study- report numbers in each exposure category, or summary measures of exposure. Cross-sectional study- report numbers of outcome events or summary measures. | 20 / 21 |
| 16. a) Give unadjusted estimates and, if applicable, confounder-adjusted estimates and their precision (e.g. 95% confidence interval). Make clear which confounders were adjusted for and why they were included. | 21 / 21 |
| 16. b) Report category boundaries when continuous variables were categorised. | 21 / 21 |
| 16. c) If relevant, consider, translating estimates of relative risk into absolute risk for a meaningful time period. | n/a |
| 17. Report other analyses done- e.g. analyses of subgroups and interactions, and sensitivity analyses. | 21 / 21 |
| 18. Summarise key results with reference to study objectives. | 21 / 21 |
| 19. Discuss limitations of the study, taking into account sources of potential bias or imprecision. Discuss both direction and magnitude of potential bias. | 11 / 21 |
| 20. Give a cautious overall interpretation of results considering objectives, limitations multiplicity of analyses, results from similar studies and other relevant evidence. | 19 / 21 |
| 21. Discuss generalisability (external validity) of the study results. | 11 / 21 |
| 22. Give the sources of funding and the role of the funders for the present study and, if applicable, for the original study on which the present article is based. | 3 / 21 |
| Risk of Bias Assessment Tool | |
| Item | **Number of studies deemed low risk of bias for item / total number of studies** |
| 1. Is the study population representative of the general adult population with TBI or spinal injury? | 10 / 21 |
| 1. Is an appropriate sample frame used, OR, whole population of interest included? | 21 / 21 |
| 1. Was sample size adequate? | 18 / 21 |
| 1. Was there significant missing data? | 7 / 21 |
| 1. Was an acceptable definition of TBI used? | 15 / 21 |
| 1. Was an acceptable definition of spinal injury used? | 19 / 21 |
| 1. Was an objective, standardised protocol used to identify spinal injury or TBI in all patients? | 9 / 21 |
| 1. Was the prevalence period appropriate? | 21 / 21 |
| 1. Is the study population described in detail? | 20 / 21 |
| 1. Is numerator and denominator for the prevalence calculation appropriate? | 20 / 21 |
| 1. Was appropriate statistical analysis conducted? | 17 / 21 |
